# Disability prevalence, disclosure, and accommodation use in pediatric residency: results from a pilot study

**DOI:** 10.1080/10872981.2026.2724652

**Published:** 2026-08-31

**Authors:** Bridget Boyd, Amy Rule, Oriaku A. Kas-Osoka, Alan Schwartz, Christopher J. Moreland, Liz Abernathey, Lisa M. Meeks

**Affiliations:** a Division of General Pediatrics, Stritch School of Medicine, Loyola University Medical Center, Chicago, IL, USA; b Divisions of Neonatology and Hospital Medicine Department of Pediatrics, Emory University School of Medicine, Atlanta, GA, USA; c Children’s Healthcare of Atlanta, Atlanta, GA, USA; d Department of Pediatrics, Division of Adolescent Medicine, University of Arkansas for Medical Sciences, Little Rock, AR, USA; e Departments of Medical Education and Pediatrics, University of Illinois Chicago, Chicago, IL, USA; f Division of Hospital Medicine, The University of Texas at Austin Dell Medical School, Austin, TX, USA; g Department of Pediatrics, Seattle Children’s Hospital/University of Washington, Seattle, WA, USA; h Department of Medical Education, The University of Illinois College of Medicine, Chicago, IL, USA; i The Department of Family Medicine, University of Michigan School of Medicine, Ann Arbor, MI, USA; j Northwestern Pritzker School of Law, Chicago, IL, USA

**Keywords:** Disability, residency, pediatrics, accommodations, disclosure

## Abstract

**Introduction:**

Disability inclusion in residency is essential to building a diverse and equitable physician workforce. Recent Accreditation Council for Graduate Medical Education requirements mandate that programs maintain formal disability policies and provide reasonable accommodations. However, residents with disabilities continue to face barriers, including stigma, complex documentation processes, and limited institutional infrastructure, challenges that differ from medical school settings. Specialty-specific data are necessary to identify gaps in inclusion, especially given that national data show higher rates of disability among trainees entering pediatrics. This pilot study reports the prevalence and accommodation practices of four pediatric residency programs.

**Methods:**

In collaboration with the Association of Pediatric Program Directors Longitudinal Educational Assessment Research Network, we conducted the first pilot survey examining disability prevalence, disclosure patterns, and accommodation use among pediatric residents. The survey was distributed to four programs between April and October 2024. Data were analyzed descriptively, with Fisher’s exact tests used to examine group differences.

**Results:**

Of the sixty-nine respondents, 36% self-identified as having a disability, more than triple the national average for residents with disabilities. ADHD, chronic health conditions, and psychological disabilities were the most reported. Fear of stigma and lack of documentation issues emerged as barriers to requesting accommodations. First-generation college graduates were significantly more likely to be unsure of their disability status.

**Discussion:**

This pilot study provides preliminary and important insights into disability prevalence, disclosure, disability uncertainty, and accommodation use in pediatric residency programs. While these findings suggest encouraging rates of accommodation receipt among disabled residents, they also highlight stigma and uncertainty around disability identity and disclosure. Findings should be interpreted in the context of the small sample size, the self-selected participation of programs, and potential response bias.

## Introduction

Disability is increasingly recognised as an important dimension of diversity within the physician workforce and an essential component of equitable medical education [1–5]. Over the past decade, accrediting bodies [6–8], medical education and physician associations [9–12], and specialty societies [13,14], have increasingly called for the inclusion of physicians and trainees with disabilities, recognising that disability representation strengthens the physician workforce, enhances care for patients with disabilities, broadens perspectives within healthcare teams, and contributes to efforts to reduce health disparities [6–14]. In the United States, the ACGME requires sponsoring institutions and residency programmes to maintain disability accommodation policies and provide reasonable accommodations to residents with disabilities. [6,7] However, implementation of these regulations remains uneven [11,15,16], and residents and physicians with disabilities continue to report unmet accommodation needs [17–19], which have been associated with increased depressive symptoms, burnout, medical errors, and workforce attrition. [18,19] Despite growing attention to the topic of disability inclusion, association guidance and accreditation requirements, the inclusion of residents with disabilities in post graduate medical education (GME) continues to lag behind other equity efforts.

Several barriers complicate this landscape. Research across medical specialties has identified persistent challenges including fear of stigma and bias [17,20–26], and career-related repercussions associated with disability disclosure including institutional ableism [26,27], lack of specialised disability support [28–30], poorly defined accommodation and disclosure processes [11,17,20,22,24,25,30] limited understanding of legal obligations and accommodation practices [11,29] and inconsistent approaches to accommodation decision-making [24,30]. Residents may also face repeated disclosure requirements and bear substantial responsibility for navigating and managing the accommodation process, often requiring ongoing self-advocacy to obtain and maintain access [31]. Together, these barriers may contribute to non-disclosure, unmet accommodation needs, and reduced programme access among residents with disabilities.

Recent scholarship and advocacy by physicians with disabilities in paediatrics have called for structural reform, improved data collection, and increased awareness in GME [32,33]. Studies on disability representation in GME suggest that between 4-20% of residents identify as having a disability [17,20,24,25], though prevalence may be underestimated due to underreporting and differences in how disability is understood. Additionally, specialty-specific studies have begun to characterise disability prevalence, disclosure, and access to accommodations in fields such as Internal Medicine [20], Surgery [24], Physical Medicine and Rehabilitation [25], and Emergency Medicine [34]. However, relatively little is known about the experiences of disabled residents in Paediatrics, despite National Residency Match Programme data indicating that the specialty has one of the highest proportions of matched applicants with disabilities [35] and the advocacy of current paediatric scholars [32,33,36,37].

The number of disabled physicians in the workforce, currently hovering at 3%, has not kept pace with the representation of trainees [38]. Specialty-specific data may be critical to identifying drivers of the training-to-practice decline. Such data may provide insight into how disability experiences vary by specialty, disability type, and demographic background, helping identify groups that may be particularly impacted by issues of non-disclosure and unmet accommodation needs, and who may be vulnerable to attrition. To address this gap, the authors partnered with the Association of Paediatric Programme Directors (APPD) and the APPD Longitudinal Educational Assessment Research Network (LEARN) to conduct the first pilot study examining disability prevalence, disclosure patterns, and accommodation use among paediatric residents.

## Methods

### Survey development

The survey incorporated disability-related questions previously used in the Association of American Medical Colleges (AAMC) Year Two Questionnaire (Y2Q) and Graduation Questionnaire (GQ) [22], which have served as the foundation for much of the disability prevalence and accommodation research conducted in U.S. medical education. These same disability items have subsequently been adopted by and used in studies from the University of Michigan Intern Health Study [17,18,39], and used in studies from the AAMC National Sample Survey of Physicians (NSSP), [19,28,40] and the American College of Physicians In-Training Examination (ITE) [20], allowing comparisons across the undergraduate medical education, graduate medical education, and physician workforce continuum. Questions assessing disability status, disability type, receipt of accommodations, and reasons accommodations were adopted directly from previously published surveys [19,20,22,39,41].

The study team and Association of Pediatric Program Directors Longitudinal Educational Assessment Research Network (APPD LEARN) survey methodologists reviewed and approved the survey. Reviewers evaluated item clarity, relevance to paediatric residency training, and alignment with the study objectives. The final survey was reviewed by authors with expertise in disability research, graduate medical education, survey methodology, and disability inclusion in medical education, who are also clinicians and researchers with disabilities (LMM, CJM, and AR). The complete survey instrument is available in Supplemental Appendix 1.

### Disability status

Disability status was determined through self-identification. Participants were asked, “Are you a person with a disability (e.g. ADHD, learning, psychological, chronic health, mobility, hearing, vision, etc.)?” and could respond “Yes,” “No,” or “I don’t know.” Participants responding “Yes” or “I don’t know” were subsequently asked to identify one or more disability categories, including attention-deficit/hyperactivity disorder (ADHD), chronic health disability, deaf or hard of hearing, learning disability, mobility disability, psychological disability, visual disability, or another disability category. No diagnostic verification was requested, and no formal disability definitions were provided, consistent with prior national studies utilising these measures.

### Accommodation use and reasons for non-use

Accommodation use was assessed using the question, “Has your residency programme provided accommodations for your disability?” Residents who reported not receiving accommodations were asked to identify reasons, including denied requests, pending accommodation determinations, lack of perceived need, fear of stigma or bias, lack of supporting documentation, and unclear institutional accommodation processes.

### Participants and procedures

This study was conducted as a multi-site pilot project through APPD LEARN. A total of 170 paediatric residency programmes participating in APPD LEARN were invited to participate. Interested programmes were required to obtain approval or exemption from their local Institutional Review Board (IRB) prior to participation. Although multiple programmes expressed interest in joining the study, only four programmes were able to secure the necessary IRB approvals within the study timeframe and subsequently participated in the pilot. Participating programmes distributed the survey electronically to residents between April and October 2024 using a secure online survey platform. The final sample consisted of sixty-nine resident respondents across four paediatric residency programmes.

### Statistical analysis

Given the exploratory nature of this pilot study and the study aim of examining disability prevalence, disclosure patterns, and accommodation use among paediatric residents, analyses were primarily descriptive. Frequencies and proportions were calculated for categorical variables. Associations between disability status and participant demographic characteristics were examined using Fisher’s exact tests due to the small sample size and low expected cell counts. All analyses were conducted using R version 4.2 (R Foundation for Statistical Computing, Vienna, Austria). The Loyola University Medical Centre Institutional Review Board determined this study to be exempt.

## Results

### Disability demographics

Sixty-nine residents responded to the survey. Twenty-five (36%) self-identified as having a disability; 41 (59%) did not identify as having a disability, and 3 (4%) reported being unsure whether they had a disability (Table 1). Self-disclosed disability status was not associated with training year, gender, race, religion, or sexual orientation. However, first-generation college graduates were more likely to report uncertainty regarding disability status than their non-first-generation peers (22% vs. 2%; Fisher's exact *p* = 0.043; OR = 15.6, 95% CI 1.1−496).

**Table 1.** Demographic and training characteristics of responding residents.

Self-identified disability status	Yes (*n* = 25)	No (*n* = 41)	Unsure (*n* = 3)	Total (*n* = 69)
**Year of training**				
PGY-1	10 (40%)	20 (49%)	1 (33%)	31 (45%)
PGY-2	5 (20%)	10 (24%)	1 (33%)	16 (23%)
PGY-3	10 (40%)	10 (24%)	1 (33%)	21 (30%)
PGY-4	0 (0%)	1 (2%)	0 (0%)	1 (1%)
USMG/IMG				
USMG	20 (80%)	34 (83%)	2 (67%)	56 (81%)
IMG	5 (20%)	7 (17%)	1 (33%)	13 (19%)
**First generation college graduate**				
Yes	3 (12%)	4 (10%)	2 (67%)	9 (13%)
No	22 (88%)	37 (90%)	1 (33%)	60 (87%)
**Gender**				
(Missing)	0	1	0	1
Female	19 (76%)	28 (70%)	2 (67%)	49 (72%)
Male	6 (24%)	12 (30%)	1 (33%)	19 (28%)
**Race/ethnicity**				
(Missing)	1	1	0	2
Underrepresented in medicine (URiM)^*^	6 (25%)	9 (22%)	0 (0%)	15 (22%)
Non-URiM Asian	3 (12%)	12 (30%)	2 (67%)	17 (25%)
Non-URiM Non-Asian White	15 (62%)	19 (48%)	1 (33%)	35 (52%)
**Sexual orientation**				
(Missing)	2	4	0	6
Bisexual/Pansexual	5 (22%)	0 (0%)	0 (0%)	5 (8%)
Gay	0 (0%)	1 (3%)	0 (0%)	1 (2%)
Queer	0 (0%)	1 (3%)	0 (0%)	1 (2%)
Straight	18 (78%)	35 (95%)	3 (100%)	56 (89%)
**Religion**				
(Missing)	2	4	0	6
Christian	13 (57%)	17 (46%)	0 (0%)	30 (48%)
Other religion, atheist, or agnostic	10 (43%)	20 (54%)	3 (100%)	33 (52%)

^*^ Underrepresented in medicine is defined as Black/African American, American Indian/Alaska Native, Hispanic/Latino/Spanish Origin, Native Hawaiian/Pacific Islander.

### Category of disability

Residents reported a total of 34 disability categories. 13 with ADHD who said “yes” to having a disability, and 1 with ADHD who was “unsure”, 8 chronic health disabilities, 6 with psychological disabilities who said “yes”, and 1 who was “unsure”, 2 deaf or hard of hearing (DHOH), 2 mobility disabilities, and 1 with “other” disability who said “yes”, and 1 who was “unsure”. One respondent reported a learning disability, and one reported a visual disability (Table 2). Because respondents could select more than one disability category, categories were not mutually exclusive.

**Table 2.** Self-reported disability-related characteristics for learners who endorsed having a disability.

Self-Reported Disability Category	Number (%) of residents (*n* = 25)
ADHD	13 (52%)
Chronic health disability	8 (32%)
Psychological disability	6 (24%)
Hearing disability	2 (8%)
Mobility disability	2 (8%)
Other disability	1 (4%)
Learning disability	1 (4%)
Visual disability	1 (4%)
**Accommodations**	
Not needed	12 (48%)
Needed and received	8 (32%)
Needed and non-received	5 (20%)
**Reported reasons for non-accommodation when needed**	
Requested but did not receive accommodations because request was denied	1 (4%)
Did not request accommodations due to lack of documentation	1 (4%)
Did not request accommodations due to fear of stigma	3 (12%)

Note: Disability categories total more than 100% as residents could report more than one disability.

### Accommodation use

Among residents who identified as having a disability, 12 (48%) reported not requiring accommodations from their residency programmes, 8 (32%) reported receiving accommodations, and 5 (20%) reported not having accommodations in place (Table 2). Among the 5 residents without accommodations, 1 reported a denied request, 1 reported lacking documentation to support a request, and 3 reported not requesting accommodations due to concerns about stigma or bias.

## Discussion

In this pilot study of paediatric residency programmes, 36% of responding residents self-identified as having a disability. Consistent with previous research, the most commonly reported disabilities were ADHD, chronic health conditions, and psychological disabilities [17,20,24,25,34]. Prevalence of disability in this sample was higher than estimates reported in national studies of graduate medical education (11.9%) [17], internal medicine (9.5%) [20], emergency medicine (4%) [34], physical medicine and rehabilitation (9.3%) [25] and general surgery (10.6-20.2% depending on category of disability) [24]. While the reasons for greater representation of disability in this sample are uncertain, it may reflect characteristics of the participating programmes, which were self-selected and may have fostered environments that support disability disclosure and inclusion. Response bias may also have contributed, as residents with disabilities may have been more likely to participate in a study focused on disability experiences.

Notably, residents who were first-generation college graduates were significantly more likely to report uncertainty regarding disability status. This uncertainty may stem from reduced exposure to disability-related language or formal diagnostic processes, particularly for non-apparent disabilities. It may also reflect broader uncertainty about what constitutes a disability within educational and professional settings. Previous research with medical students has demonstrated that learners who report being “unsure” whether they have a disability experience higher rates of burnout than both students who identify as disabled and those who do not identify as disabled [42]. Additionally, first-generation learners are less likely to receive the accommodations needed in clinical training environments [43].

Importantly, nearly half of residents who identified as having a disability reported that they did not require accommodations from their residency programme. Several explanations may account for this finding. Some residents may effectively manage their disabilities without formal accommodations, while others may benefit from informal supports, universally designed educational practices, or training environments that reduce the need for individualised accommodations. However, prior studies suggest that some learners and residents with disabilities choose not to pursue formal accommodations because of concerns regarding stigma, bias, administrative burden, or uncertainty about whether accommodations are warranted. [17,20,24,25]. Accordingly, the absence of formal accommodations should not necessarily be interpreted as the absence of disability-related needs.

Among residents who reported a need for accommodations, most received them, a promising finding given previously reported barriers to accommodation access in residency and uneven implementation of disability-related policies [5,15]. However, in our sample, reported fear of stigma and lack of documentation remained important barriers, mirroring findings from previous studies [17,20,24,25]. The continued reporting of stigma-related concerns underscores the importance of transparent accommodation processes and training environments that support disclosure and help-seeking behaviours. This remains particularly important given evidence linking unmet accommodation needs with burnout, increased depressive symptoms, and increased risk of medical error [18].

This study has several limitations. The self-selection of participating programmes may limit the generalisability of these findings. Programmes with existing disability-related initiatives, infrastructure, or interest may have been more likely to participate. In addition, the small sample size and limited number of participating programmes reduced statistical power and may have limited our ability to detect meaningful differences between groups. Additionally, disability status was self-reported, and no formal definition was provided to respondents. As a result, participants may have interpreted what constitutes a disability differently. Disability stigma may also contribute to underreporting. Finally, because only four residency programmes participated, programme-level characteristics could not be reported without increasing the risk of institutional identification. Despite these limitations, this pilot study provides important specialty-specific data regarding disability prevalence, disability-status uncertainty, disclosure, and accommodation use in paediatric residency training and highlights priority areas for future research.

Together, these findings suggest that further study is warranted to better understand the true prevalence of disability, disclosure patterns within paediatric training, and the factors contributing to disability-status uncertainty, which may represent an underrecognized source of stress and an important opportunity for intervention. Paediatrics, a specialty rooted in empathy, equity, and advocacy, is uniquely positioned to lead efforts to advance disability inclusion PED [44]. Although compliance with ACGME requirements for disability policies and accommodations is essential, it is unlikely to be sufficient on its own. Programmes must also foster learning environments that support disclosure, reduce stigma, and recognise disability as an important dimension of diversity within the physician workforce. By doing so, residency programmes can improve resident well-being, retention, and educational outcomes while cultivating a physician workforce better prepared to care for children and families with disabilities.

## Conclusions

This multi-site pilot study provides preliminary and important insights into disability prevalence, disability-status uncertainty, disclosure, and accommodation use among paediatric residents. While the findings suggest encouraging rates of accommodation receipt among residents who reported needing accommodations, they also highlight the continued influence of stigma and uncertainty around disability identity and disclosure. Adherence to ACGME-required disability policies, coupled with deliberate efforts to reduce stigma, improve disability literacy, and create supportive training environments, will be essential to ensuring that paediatric residents with disabilities can fully participate and thrive in residency training.

## Supplementary Material

Supplementary Material LimeService_-_Your_online_survey_service.pdf

## Data Availability

Due to the small sample size and the risk of participant or institutional identification, deidentified data are not publicly available. Requests for limited deidentified data may be considered by the corresponding author on a case-by-case basis, subject to institutional approvals and protections for participant confidentiality.
