# Supplementary material for: Disability prevalence, disclosure, and accommodation use in pediatric residency: results from a pilot study
Source: Med Educ Online. 2026 Aug 31;31(1):2724652. doi: 10.1080/10872981.2026.2724652 (PMC13532363; doi:10.1080/10872981.2026.2724652)
Supplement: Supplementary file 1 [file ZMEO_A_2724652_SM6474.pdf]

Disability Prevalence, Accommodations and Belonging in Pediatric Residents (Residents)

Welcome to the Disability Prevalence, Accommodations, and Belonging in Pediatric Residents!

The purpose of this study is to obtain data about disability prevalence, accommodations, and belonging in pediatric residents who identify as having a disability. You are being asked to take part in a research study because you are a pediatric trainee in a categorical pediatric program.

Your involvement in this research study is completely voluntary and will not in any way affect your academic and/or employment standing with your respective program. Your responses will be de-identified and will not be linked to your name. The results will be presented in aggregate and NOT by training programs to further protect the identity of participants. APPD LEARN will receive your de-identified responses, and the de-identified, anonymous responses will be forwarded to the study team listed below for data analysis.

This study has been found exempt by the IRB at Loyola University Medical Center.

If you have questions or concerns about this study please contact the PI, Dr. Bridget Boyd at brboyd@lumc.edu

Principal Investigators:

Bridget Boyd, MD, Loyola University Medical Center

Amy Rule, MD, MPH, Emory School of Medicine

Oriaku Kas-Osaka, MD, M.Ed., Arkansas Children’s Hospital Little Rock

Christopher Moore, MD, University of Texas at Austin

Lisa Meeks, PhD, University of Michigan Ann Arbor

There are 21 questions in this survey.

Initial Group

{regexMatch('/^[0-9]{10,11}$/',learnid)} *

Personal Demographics

Please select your current training year. *

Choose one of the following answers

Please choose **only one** of the following:

- ◯ PGY-1
- ◯ PGY-2
- ◯ PGY-3
- ◯ PGY-4
- ◯ PGY-5
- ◯ Other ______

Which of the following describes the school where you obtained your medical degree? *

Choose one of the following answers

Please choose **only one** of the following:

- ◯ I graduated from a medical school located in the U.S. or Canada
- ◯ I graduated from a medical school located outside the U.S. or Canada

If you graduated from a medical school outside of the U.S. or Canada, were you *

Only answer this question if the following conditions are met:
Answer was 'I graduated from a medical school located outside the U.S. or Canada' at question ' [countrymeddegree]' (Which of the following describes the school where you obtained your medical degree?)

Choose one of the following answers
Please choose **only one** of the following:

- ◯ Born within the U.S.A. (including its territories)
- ◯ Born outside of the U.S.A.
- ◯ Prefer not to answer
- ◯ Other ______

What best describes your current training program? *

Choose one of the following answers
Please choose **only one** of the following:

- ◯ Categorical pediatric residency
- ◯ Combined pediatric and another specialty residency (ex: Med-Peds, Child neuro, etc)
- ◯ Other ______

Are you first generation college graduate? *

Choose one of the following answers
Please choose **only one** of the following:

- ◯ Yes
- ◯ No

What is your current gender identity? (Select all that apply.) *

Please choose **all** that apply:

- ☐ Female
- ☐ Male
- ☐ Male-to-Female (MTF)/Transgender Female/Trans Woman
- ☐ Female-to-Male (FTM)/Transgender Male/Trans Man
- ☐ Non-Binary
- ☐ Prefer not to answer
- ☐ Other: ____________

Which one or more of the following would you say is your race/ethnicity? (Select all that apply.) *

Select all that apply

Please choose **all** that apply:

- [ ] American Indian or Alaska Native
- [ ] Asian
- [ ] Black or African-American
- [ ] Hispanic, Latino, or Spanish Origin
- [ ] Native Hawaiian or Other Pacific Islander
- [ ] White
- [ ] Prefer not to answer
- [ ] Other: ____________

Which of the following best represents your sexual orientation? *

Select all that apply

Please choose **all** that apply:

- [ ] Bisexual/Pansexual
- [ ] Gay
- [ ] Heterosexual
- [ ] Lesbian
- [ ] Prefer not to answer
- [ ] Other: ____________

Please indicate your religion or spiritual preference. *

Select all that apply

Please choose **all** that apply:

- ☐ Agnostic
- ☐ Atheist
- ☐ Christianity
- ☐ Hinduism
- ☐ Islam
- ☐ Judaism
- ☐ Prefer not to answer
- ☐ Other: ____________

Eligibility

Are you a person with a disability (e.g., ADHD, learning, psychological, chronic health, mobility, hearing, vision, etc? *

Choose one of the following answers

Please choose **only one** of the following:

- ○ Yes
- ○ No
- ○ I don't know

Disability Core Questions

Which of the following best describes your disability? If you have more than one type, select all that apply.
*

Only answer this question if the following conditions are met:
Answer was 'I don't know' *or* 'Yes' at question ' [eligibility]' (Are you a person with a disability (e.g., ADHD, learning, psychological, chronic health, mobility, hearing, vision, etc?)

Select all that apply
Please choose **all** that apply:

- ☐ Attention-deficit/hyperactivity disorder
- ☐ Chronic health disability
- ☐ Deaf or hard of hearing
- ☐ Learning disability
- ☐ Mobility disability
- ☐ Psychological disability
- ☐ Visual disability
- ☐ Other: ____________

Has your residency program provided accommodations for your disability? *

Only answer this question if the following conditions are met:
Answer was 'Yes' *or* 'I don't know' at question ' [eligibility]' (Are you a person with a disability (e.g., ADHD, learning, psychological, chronic health, mobility, hearing, vision, etc?)

Choose one of the following answers
Please choose **only one** of the following:

- ○ Yes
- ○ No

Which of the following best describes why your residency program did not or has not provided accommodations? *

Only answer this question if the following conditions are met:
Answer was 'Yes' *or* 'I don't know' at question ' [eligibility]' (Are you a person with a disability (e.g., ADHD, learning, psychological, chronic health, mobility, hearing, vision, etc?) *and* Answer was 'No' at question ' [accomodations]' (Has your residency program provided accommodations for your disability?)

Select all that apply
Please choose **all** that apply:

- ☐ My request for accommodations was denied
- ☐ My request for accommodations is under review
- ☐ I have not requested accommodations because I feel I do not need accommodations
- ☐ I have not requested accommodations because I fear stigma or bias
- ☐ I do not have documentation to support my request
- ☐ My institution does not have a clear process for requesting accommodations
- ☐ Other: ____________

Expanded Accommodations Questions

After you formally requested accommodations for your residency training, how long did it take for your program to either approve or provide accommodations? *

Only answer this question if the following conditions are met:
Answer was 'Yes' *or* 'I don't know' at question ' [eligibility]' (Are you a person with a disability (e.g., ADHD, learning, psychological, chronic health, mobility, hearing, vision, etc?) *and* Answer was 'Yes' at question ' [accomodations]' (Has your residency program provided accommodations for your disability?)

Choose one of the following answers
Please choose **only one** of the following:

- <1 week
- 1-4 Weeks
- 5-8 weeks
- > 8 weeks
- Other ____________

How many hours did you spend in total on the accommodations request process? (could include things like meeting with program directors or other staff, attending medical appointments, providing documentation, etc). *

Only answer this question if the following conditions are met:
Answer was 'Yes' *or* 'I don't know' at question ' [eligibility]' (Are you a person with a disability (e.g., ADHD, learning, psychological, chronic health, mobility, hearing, vision, etc?)

Choose one of the following answers
Please choose **only one** of the following:

- ◯ less than 1 hour
- ◯ 1-8 hours
- ◯ 9-16 hours
- ◯ 17-24 hours
- ◯ Other ________

If your program provides care at multiple settings, did you have to repeat the accommodations request process yourself at each training location or facility? *

Only answer this question if the following conditions are met:
Answer was 'Yes' *or* 'I don't know' at question ' [eligibility]' (Are you a person with a disability (e.g., ADHD, learning, psychological, chronic health, mobility, hearing, vision, etc?)

Please choose **only one** of the following:

- ◯ Yes
- ◯ No

Is there anything else you would like to share regarding your experience with disclosure and/or the accommodations process at your training program? *

Only answer this question if the following conditions are met:
Answer was 'Yes' *or* 'I don't know' at question ' [eligibility]' (Are you a person with a disability (e.g., ADHD, learning, psychological, chronic health, mobility, hearing, vision, etc?)

Please write your answer here:

Belonging Questions

How true do you feel the following statements are about you during the past two weeks at work? *

Only answer this question if the following conditions are met:
Answer was 'Yes' *or* 'I don't know' at question ' [eligibility]' (Are you a person with a disability (e.g., ADHD, learning, psychological, chronic health, mobility, hearing, vision, etc?)

Please choose the appropriate response for each item:

	Not at all true	Somewhat true	Moderately true	Very true	Completely true
**I feel happy at work**	○	○	○	○	○
**I feel worthwhile at work**	○	○	○	○	○
**My work is satisfying to me**	○	○	○	○	○
**I feel in control when dealing with difficult problems at work**	○	○	○	○	○
**My work is meaningful to me**	○	○	○	○	○
**I'm contributing professionally (i.e. patient care, teaching, research, and leadership)**	○	○	○	○	○

How frequently have you felt the following in the past 2 weeks?

During the past two weeks, I have felt...

*

Only answer this question if the following conditions are met:
Answer was 'Yes' *or* 'I don't know' at question ' [eligibility]' (Are you a person with a disability (e.g., ADHD, learning, psychological, chronic health, mobility, hearing, vision, etc?)

Please choose the appropriate response for each item:

	Not at all	Very little	Moderately	A lot	Extremely
**A sense of dread when I think about the work I have to do**	○	○	○	○	○
**Physically exhausted at work**	○	○	○	○	○
**Lacking enthusiasm at work**	○	○	○	○	○
**Emotionally exhausted at work**	○	○	○	○	○

During the past two weeks, my job has contributed to me feeling... *

Only answer this question if the following conditions are met:
Answer was 'Yes' *or* 'I don't know' at question ' [eligibility]' (Are you a person with a disability (e.g., ADHD, learning, psychological, chronic health, mobility, hearing, vision, etc?)

Please choose the appropriate response for each item:

	Not at all	Very little	Moderately	A lot	Extremely
**Less empathetic with my patients**	○	○	○	○	○
**Less empathetic with my colleagues**	○	○	○	○	○
**Less sensitive to others' feelings/emotions**	○	○	○	○	○
**Less interested in talking with my patients**	○	○	○	○	○
**Less connected with my patients**	○	○	○	○	○
**Less connected with my colleagues**	○	○	○	○	○

Thank you for your participation!
01-16-2025 – 09:49
Submit your survey.
Thank you for completing this survey.
